# VIRDENTOPSY: Virtual Dental Autopsy and Remote Forensic Odontology Evaluation

**DOI:** 10.3390/dj9090102

**Published:** 2021-09-05

**Authors:** Emilio Nuzzolese

**Affiliations:** Human Identification Laboratory, Section of Legal Medicine, University of Turin, 10124 Torino, Italy; emilio.nuzzolese@unito.it; Tel.: +39-011-6705969

**Keywords:** virdentopsy, virtual dental autopsy, autopsy imaging, human identification, dental autopsy, humanitarian forensic odontology, forensic odontology

## Abstract

The identification of human remains relies on the comparison of post-mortem data, collected during the autopsy, with the ante-mortem data gathered from the missing persons’ reports. DNA, fingerprints, and dental data are considered primary identifiers and are usually collected during any human identification process. Post-mortem dental data should be collected and analyzed by forensic odontologists, as a dental autopsy must not be confused with a dental examination. The virdentopsy project was inaugurated in 2020, during the COVID-19 pandemic, to allow the correct process of human remains by collecting dental data from teeth and jaws, which was then transmitted to forensic odontologists remotely for an expert opinion to achieve a generic profile of the unidentified human remains. The post-mortem dental biography is paramount to narrow the search for compatible missing persons but requires knowledge and experience of forensic odontologists. The virdentopsy process uses radiographic imaging (periapical X-rays, CT scans, panoramics), 2D/3D photos and video recording, photogrammetry documentation, 3D scanning, and live streaming where possible. This registered term was created by merging the terms “virtual” and “dental autopsy” but with no commercial benefits. The proposed process combines research topics under the field of the human rights of the dead and humanitarian forensic odontology services. It should enhance and accelerate the human identification process of the deceased, age estimation of the living, analysis of panoramic X-ray images, and be an educational tool for remote live training in forensic odontology and anatomy of skulls. This paper presents an overview of the virdentopsy process in the field of forensic odontology as a remote consultation as well as an educational tool for undergraduates and postgraduates.

## 1. Introduction

The identification of the deceased requires the involvement of several forensic experts, depending on the status of the remains. There are several methods to evaluate the evidence used to identify human remains: visual recognition; fingerprints; DNA; dental data [1]; and physical evidence (e.g., tattoos, scars, and surgical implants) [2]. The visual method is not recommended because of subjective factors and the high risk of misidentification. It can, however, lead to a single-case presumptive identification when combined with other evidence, such as personal belongings or eye-witness reports [3]. Nevertheless, it is internationally accepted that the most reliable method for identification is the collection of primary identifiers: friction ridge analysis, forensic odontology, and DNA [4]. 

The positive identification allows surviving family members to start the grieving process, and the death certificate following identification allows them to set insurance, legal, business, and personal affairs in order. Giving a name and an identity to unidentified human remains is also fundamental for the respect of the human rights of the dead and a dignified disposition of human remains [5,6].

The collection and analysis of dental data for the human identification process is paramount in comparing the data from the unidentified remains to the reported missing persons list and achieving a preliminary list of compatible profiles. 

During the dental autopsy, forensic odontologists collect all dental and radiological evidence from teeth and jaws, as well as 3D scanning on soft tissue of the face when possible, and by analyzing this data, they estimate a generic biological profile, which is used to search for missing persons compatible with the unidentified human remains. 

This assessment includes sex, geographical origin, dental age, cheiloscopy [7,8], and other biological markers, such as oral hygiene and dietary habits, and will possibly achieve a positive identification confirming a match with ante-mortem dental data of the missing persons. The principle in the human identification process is the comparison of ante-mortem with post-mortem evidence and findings among primary identifiers: fingerprints, DNA, and dental data. The correct collection and interpretation of dental information requires experienced dentists with forensic backgrounds and knowledge in disaster victim identification (DVI). Although forensic odontology is taught in most countries through postgraduate training courses for dentists, forensic odontologists are not always available or recruited systematically in the human identification process. Furthermore, the COVID-19 pandemic has raised several safety concerns when dealing with human remains of unknown medical history [9], in caseworks as well as in hands-on forensic training courses. A possible solution is the use of teledentistry [10] in forensic odontology for remote consultation of dental autopsies, as well as a supplementary means of education and hands-on training to develop skills in odontology and learn the anatomy of the skull. 

## 2. Virtual Autopsy and Teledentistry in Forensic Odontology 

The idea of using non-invasive imaging methods to perform a virtual autopsy was presented in 2003 as a tool in forensic pathology [11] to document and analyze forensic findings in dead persons [12]. It consists of whole-body volume documentation using CT, MRI, and radiology, combined with a 3D body documentation using photogrammetry and optical scanning [13], which allows an alternative and non-invasive approach to examining dead bodies and to evaluate the cause and manner of death and any other relevant forensic findings. Applications of teledentistry in a virtual autopsy have been presented by several authors [14,15,16] but without any humanitarian goal or applications in the identification of dead migrants. Virtual autopsy has also been proposed for odontological cases [17,18] and for the identification process [19]. As mentioned above, the major concern in the identification process is the collection of primary identifiers, including dental data through a complete dental autopsy performed by one or more odontologists who are experts in forensic odontology and disaster victim identification [20]. To be effective in the identification purpose, post-mortem dental data collected during the dental autopsy should receive a proper analysis and evaluation by forensic odontologists. If they are not available onsite, it is conceivable to split the post-mortem dental data collection, performed by dentists onsite, and the forensic odontology evaluation of data collected, performed remotely by forensic odontologists. Furthermore, when the nationality of the deceased is unknown or for challenging cases, a second expert opinion could be advisable. Finally, the involvement of remote forensic odontologists available pro bono could be a supplementary means in humanitarian forensic odontology and missing and unidentified persons as well as unidentified dead migrants.

## 3. The Virtual Dental Autopsy Project (VIRDENTOPSY)

The Human Identification Laboratory and the medico-legal section of the University of Turin started a research project in 2020 based on the hypothesis that the identification process of unidentified human remains must always comply with best practices in human identification, which should always include a complete dental autopsy even when no forensic odontologists are available onsite. Furthermore, teleconsultation in medicine and dentistry, especially after the COVID-19 pandemic [21] and potential risk of infection [22], can be applied also in forensics, and specifically in the human identification process. This allows forensic pathologists to perform the autopsy without compromising on the technical inputs of forensic odontologists. 

**Figure 1:**
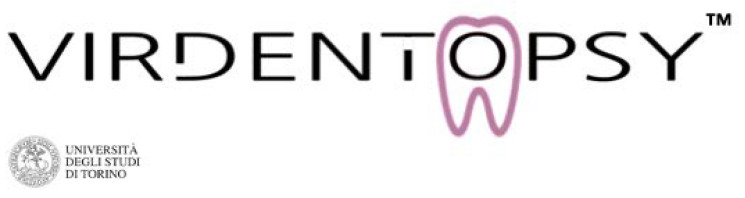
Virdentopsy™ registered brand (Class 44).

At present, there are few institutions worldwide that have recognized the feasibility of remote dental autopsy [5,23] but none are currently offering teleconsultations in forensic odontology for the purpose of human identification or considering offering this service on a humanitarian basis. The project combines research topics such as pathology, odontology, anthropology, and archeology under the umbrella of human rights of the dead and humanitarian forensic odontology [24,25,26]. The term VIRDENTOPSY merges the terms “virtual” and “dental autopsy”. It is a registered brand (Figure 1) with a dedicated website [27] in order to offer a remote forensic odontological assessment of post-mortem dental data of unidentified human remains.

**Figure 2:**
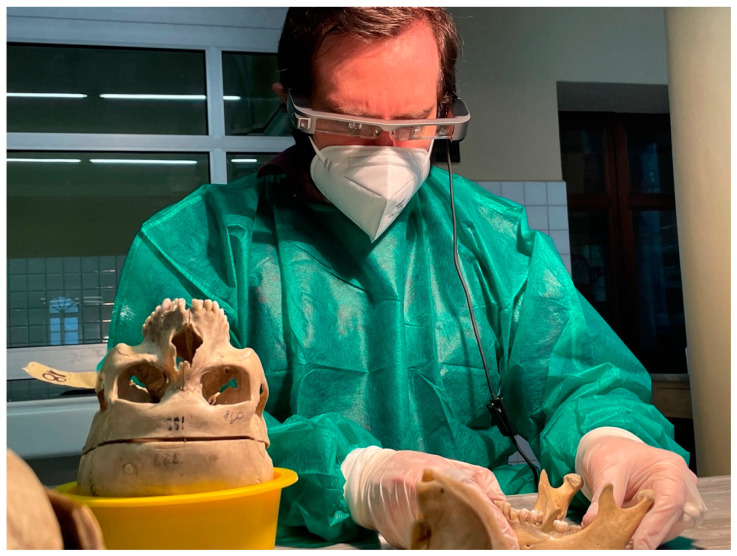
Operator using smart glasses to observe and record dental features on the mandible.

**Figure 3:**
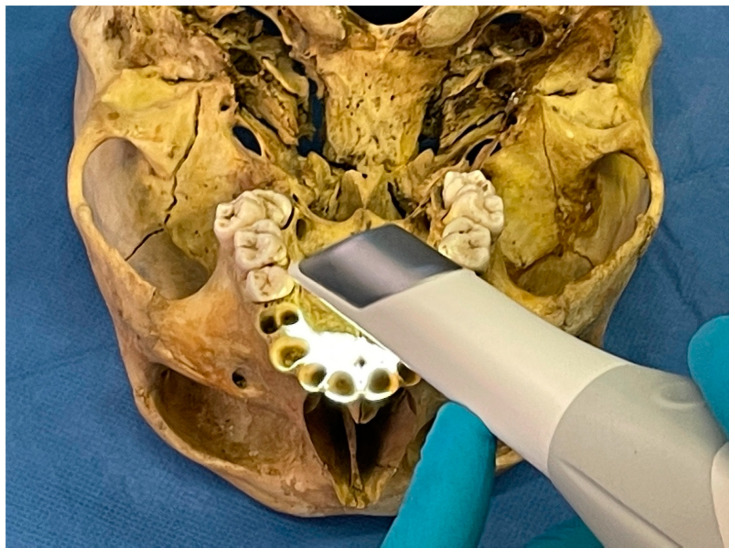
Post-mortem photogrammetry collection of upper dental arch using an intraoral scanner.

**Figure 4:**
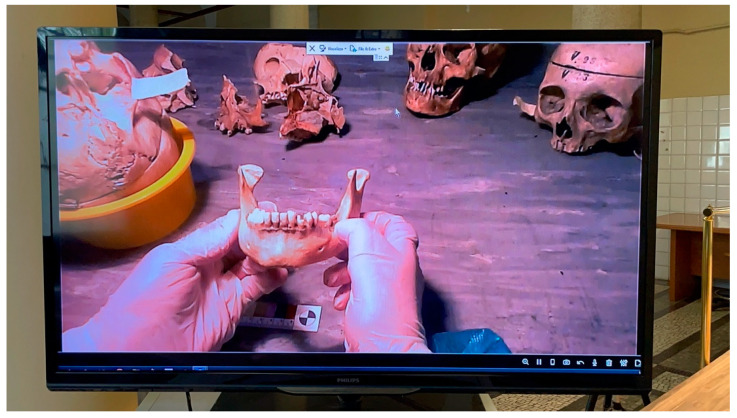
Live streaming images observed remotely from a forensic odontologist.

Virdentopsy makes provisions for the systematic collection of post-mortem dental data performed by forensic pathologists, dentists with no forensic background, dental hygienists with a forensic background, or other forensic operators authorized in the mortuary. These operators perform the dental and intraoral collection of postmortem dental data (also in livestreaming), following what is usually performed by forensic odontologists in the preliminary dental examination of human remains, which is one of the stages of a traditional dental autopsy [28]. Data can be transmitted to the human identification laboratory, where one or more forensic odontology consultants could evaluate the data received and provide charting and the dental autopsy report [28]. Provisions on the unidentified human remains consist of the following data collection:

- 2D or 3D video recording of the dental arches and oral cavity, using intraoral camera or smartphones (Figure 2).
- Photographic collection of the dental arches.
- Photogrammetry of the dental arches using an intraoral scanner (Figure 3).
- 3D scanning of jaws and skull.
- Intraoral radiographic collection using digital sensors.
- Any radiographic imaging of the skull (Panoramic images, OPG, TC scans, if available).
- Live streaming using smartphone and smart glasses (Figure 4).

By registering on the Virdentopsy website, it will be possible to choose a type of assessment, either a single unidentified human remains, or an assessment within a DVI procedure, and decide if a primary or secondary expert opinion is required. Quality control checks would be carried out on the received data. The service is remunerated or pro-bono depending on the applicant entity. 

This forensic service will also be available for age estimations of living individuals and for hands-on training sessions of forensic odontology courses.

## 4. Discussion

The identification process of any unidentified human remains must respect best standards in forensics and should always include the collection of all identifying post-mortem data. The process requires the comparison with ante-mortem data of compatible reported missing persons and a final reconciliation of data collected. In this regard, a complete dental autopsy and further peer-review evaluation by forensic odontologists of all dental findings are crucial to achieving a generic biological profile and investigation. The simple dental examination or the collection of dental data without any evaluation performed by experts in forensic odontology and disaster victim identification does not offer any concrete contribution to the process of achieving a timely and effective personal identification. When considering the migration phenomena, there is a need for a wider collection and analysis of postmortem data [28]. In Italy, the “Missing Migrants Project” estimates that there have been 17,124 migrant deaths and disappearances since 2014, and over 60% of these victims are unidentified [29,30], while the forensic dental evaluation seems limited to a dental examination without the routine involvement of experienced forensic odontologists [31]. 

Virtual autopsy applications in forensic odontology involve the collection of dental data and comparison between ante-mortem and post-mortem orthopantomograms [30]. The virdentopsy process is not only the integration of a virtual autopsy in forensic odontology practice [32,33] aEs it does not rely only on radiographic imaging and CT scanning but also on 2D and 3D video, photos, and photogrammetry documentation. Virtual autopsy allows the examination of jaws and teeth by odontologists without the need to perform a traditional dental autopsy [7]. Virdentopsy broadens the horizon of this virtual approach, involving remote odontologists from various countries without the need to be physically onsite, and could become a standard of each unidentified dead body recovered. 

The proposed procedure is a possible solution in the following scenarios:

- Forensic odontologists are not available on the mass disaster site or on the area of the recovery (single unidentified body of unknown nationality);
- Forensic odontologists cannot be recruited or afforded in the temporary morgue after a mass disaster;
- Forensic odontologists of the same nationalities as the victims involved in the disaster can offer remotely useful hints related to dental treatments and dental data interpretation;
- A second expert opinion on all post-mortem dental data collected could widen or confirm dental findings such as age, habits, and dental treatments, thus reducing bias;
- Archeological and paleopathological findings on ancient human remains could benefit from a complementary analysis by forensic odontologists, completing the evaluation study of jaws and teeth that are available;
- Reduce infectious risks, such as those faced during the COVID-19 pandemic, by reducing the number of experts involved in the autopsy room for the post-mortem data collection;
- Hands-on training in forensic odontology courses through live streaming using smart glasses and augmented reality.

The virdentopsy screening can reduce technical consultancy costs, as forensic odontologists do not need to be onsite; however, more importantly, it can speed up the process of human forensic identification and respect forensic human identification protocols through the complete collection and evaluation of all dental evidence, optimizing the use of human resources involved in a mass disaster scenario or a single unidentified human remains. The technology required is already incorporated in the latest generation’s smartphones, making this process immediately applicable and a revolutionary humanitarian forensic odontology tool in all identification processes.

## 5. Conclusions

Best practices in human identification suggest the collection of primary and secondary identifiers, including post-mortem dental data. To achieve this goal, a complete dental autopsy should always be performed by one or more odontologists who are experts in forensic odontology and disaster victim identification. The virdentopsy procedure can enhance the identification autopsy through the supplementary assessment of remote forensic odontologists, providing a dental biography and generic biological profile of the unidentified human remains. Virdentopsy allows best practices in human identification when odontologists are not available onsite or when a second expert opinion is advised and can be considered a valuable humanitarian forensic odontology tool for the missing and unidentified human remains of unknown nationality, as well as an educational resource for hands-on training in forensic odontology.
